# The prognosis of NX stage in patients with pT1 papillary thyroid cancer who underwent lobectomy

**DOI:** 10.1097/MD.0000000000033632

**Published:** 2023-05-12

**Authors:** Ha Rim Ahn, Sang Yull Kang, Hyun Jo Youn, Sung Hoo Jung

**Affiliations:** a Department of Surgery, Research Institute of Clinical Medicine, Jeonbuk National University Hospital, Jeonbuk National University and Biomedical Research Institute, Jeonju, Korea.

**Keywords:** lymph node, papillary thyroid carcinoma, prognosis, survival

## Abstract

Lymph node (LN) metastasis is known to impact the prognosis of patients with well-differentiated thyroid cancer. Herein, we aimed to determine the effect of NX stage on the prognosis of patients with papillary thyroid cancer who underwent thyroid lobectomy. We initially selected 1257 patients who underwent thyroid cancer surgery from 2012 to 2015. Of the 1257 patients, we included 556 in the analysis, excluding patients diagnosed with other types of thyroid cancer, those who underwent total or completion thyroidectomy, and those diagnosed with LN metastasis prior to surgery. The median follow-up time was 61.8 months (range: 12.3–108.9 months). After dividing the patients into N0, N1, and NX stage groups, we performed univariate and multivariate analyses. The 5-year recurrence-free survival (RFS) was analyzed using R version 3.2.5. The mean patient age was 45.0 ± 10.9 years. Of the 556 patients, 336 patients (60.4%) were diagnosed with N0 stage, 134 (24.1%) were N1 stage, and 86 (15.5%) were NX stage. Univariate and multivariate analyses were performed to identify prognostic factors for RFS. Considering gender, age, tumor size, surgery types, extrathyroidal extension, multifocality, and recurrence, no statistically significant differences were noted between the 3 groups. The 5-year RFS rates were 98.8%, 95.5%, and 97.6% for N0, N1, and NX groups, respectively, without significant differences between the 3 groups (*P* = .56). Considering the T1b stage, the 5-year RFS rates were 100%, 93.1%, and 93.7% in the N0, N1, and NX groups, respectively, with a statistically significant difference between the 3 groups (*P* = .018). Accordingly, the NX status cannot be deemed a prognostic factor for RFS in patients with papillary thyroid cancer who underwent thyroid lobectomy. However, the benefit of prophylactic central-LN dissection should be considered in patients with well-differentiated thyroid cancer diagnosed with T1b stage.

## 1. Introduction

Thyroid cancer is the ninth most common neoplasm worldwide.^[1]^ Papillary thyroid carcinoma (PTC) is the most common histological type of thyroid cancer, and the incidence of PTC has consistently grown over the past several decades.^[2]^ Recent international guidelines recommend thyroid lobectomy as the initial surgical approach in patients who meet the predetermined criteria for low-risk PTC, such as small medium-sized (T1–T2) and N0 PTC in the absence of an extrathyroidal extension.^[3]^ Furthermore, prophylactic central-lymph node dissection (CLND) is recommended for clinically lateral neck node involvement and/or advanced primary tumor (T3 or T4).^[3]^ However, the potential role of prophylactic CLND in patients with T2 or lower unilateral PTC remains controversial.

Prophylactic CLND can reduce the local recurrence rate. Moreover, it is crucial to establish the presence of lymph node (LN) metastases, as it remains an important predictor of distant metastasis and disease relapse.^[4,5]^ Conversely, it has been suggested that central compartment LNs do not impact long-term survival; hence, CLND is not recommended for reducing perioperative morbidity caused by prophylactic CLND, such as hypoparathyroidism or unintentional recurrent laryngeal nerve injury.^[6,7]^

According to the American Joint Committee on Cancer TNM system, regional LNs cannot be assessed in the NX stage.^[8]^ In a 2018 retrospective study using the Surveillance, Epidemiology, and End Results (SEER) data, cancer-specific survival did not significantly differ between NX and N1 stages; however, NX stage has been associated with poor cancer-specific survival when compared with N0 stage.^[9]^ Accordingly, in the present study, we aimed to examine the effects of NX stage on prognosis in patients who underwent thyroid lobectomy and prophylactic CLND for clinically node-negative T1 papillary thyroid cancer.

## 2. Methods

### 2.1. Patient selection

**Figure 1.**
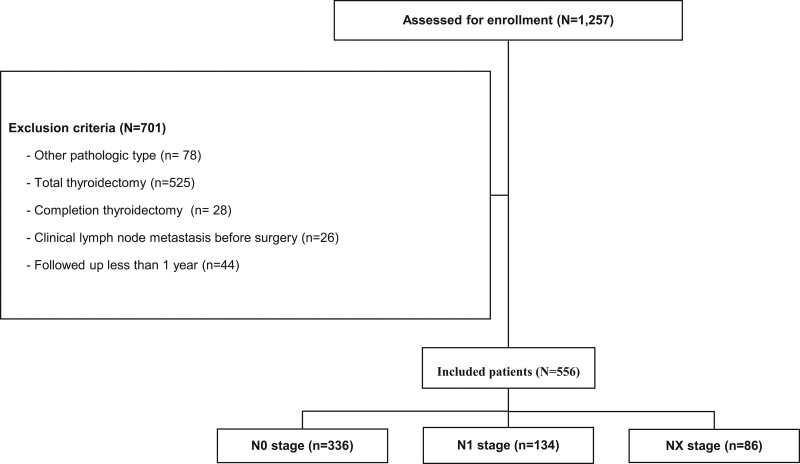
Flow chart of patients who met the inclusion/exclusion criteria for study participation.

In the present study, we retrospectively analyzed 1257 Korean patients who underwent surgery for thyroid cancer between January 1, 2012, and December 31, 2015. Considering 1257 patients, we included 556 in the analysis, excluding those diagnosed with other pathologic types of thyroid cancer (except PTC), those who underwent total thyroidectomy or completion thyroidectomy, those with cancer ˃2 cm, those clinically diagnosed with LN metastasis before surgery, and followed up for ˂1 year (Fig. 1). The median follow-up time was 61.8 months (range: 12.3–108.9 months).

### 2.2. Data collection

Patient medical records were retrospectively reviewed to determine clinicopathological data, including ethnicity, gender, age at diagnosis, tumor size, surgery type, multifocality of cancer, co-existence of Hashimoto thyroiditis, extrathyroidal extension, nodal stage, and recurrence.

### 2.3. Preoperative evaluation

All patients underwent a physical examination, ultrasonography (US), and computed tomography (CT) preoperatively. To establish a cancer diagnosis, thyroid nodules were preoperatively examined using US-guided fine needle aspiration biopsy. Considering patients diagnosed with papillary thyroid cancer, US and CT were performed preoperatively to determine tumor location, size, extrathyroidal extension, nodal metastasis, and other abnormal findings.

### 2.4. Surgery

Patients with T1, unilateral lobe PTC without clinical LN metastasis, extrathyroidal extension, and history of head or neck cancer underwent thyroid lobectomy. All patients were subjected to prophylactic CLND. During prophylactic CLND, level 6 LNs, ipsilateral to PTC (pretracheal, prelaryngeal, and paraesophageal LNs), were removed.

### 2.5. Outcomes

Based on the findings of the postoperative histopathologic examination, patients were divided into N0, NX, and N1 groups according to the pathological nodal stage. Subsequently, we examined the clinicopathological factors of each group. The presence of cervical LN metastasis and cancer stage were established according to the American Joint Committee on Cancer Cancer Staging Manual, 8th edition.^[8]^

To evaluate oncologic outcomes, all patients underwent regular ultrasonographic follow-ups. Patients with evidence of recurrence or distant metastasis were assessed using neck CT and/or positron emission tomography-CT. US-guided fine needle aspiration biopsy was performed to confirm cancer recurrence with regional LN or thyroid bed involvement.

Recurrence-free survival (RFS) was defined as the duration from the date of thyroid cancer surgery to the recurrence at a local or regional site, the occurrence of metastasis at a distant site, or a newly developed cancer at the contralateral thyroid lobe.

### 2.6. Statistical analysis

Categorical variables are described as frequencies, whereas continuous variables are reported as median and interquartile ranges. To compare categorical variables among the 3 groups, we performed Fisher exact test or Pearson chi-square test with Yates continuity correction. One-way analysis of variance (ANOVA) and Kruskal–Wallis test were used to compare continuous variables. Univariate analysis was conducted using the Kaplan–Meier method and log-rank test. Univariate and multivariate analyses were performed using the Cox proportional hazards model. Multivariate analysis was conducted if the *P* value was <0.200 in the univariate analysis. A *P* value < 0.05 was considered statistically significant. All statistical analyses were conducted using R version 3.2.5 (Vienna, Austria; http://www.R-project.org).

### 2.7. Ethical approval

All procedures performed in the present study were in accordance with the ethical standards of the Institutional Review Board of Jeonbuk National University Hospital (No. 2021-06-011) and complied with the 1964 Declaration of Helsinki and its later amendments or comparable ethical standards.

## 3. Results

### 3.1. Patient characteristics

Herein, we enrolled 556 patients, comprising 466 women (83.8%) and 90 men (16.2%). The mean patient age at the time of surgery was 45.0 years (range: 19–74 years). The mean tumor size was 0.7 cm (range: 0.1–1.9 cm), and 460 patients (82.7%) had papillary thyroid microcarcinoma (PTMC) ˂1 cm in size. Open thyroid lobectomy was performed in 470 patients (84.5%), 190 patients (34.2%) had Hashimoto thyroiditis, and 208 (37.4%) exhibited extrathyroidal extension. Of these 556 patients, 336 patients (60.4%) were diagnosed with the pathological N0 stage (N0 group), 134 (24.1%) with N1 stage (N1 group), and 86 patients (15.5%) with NX stage (NX group). Recurrence occurred in 16 (2.9%) out of 556 patients (Table 1).

**Table 1:** Clinicopathologic characteristics of patients.

Characteristics	Value (n = 556)
Gender	
Female	466 (83.8%)
Male	90 (16.2%)
Age (yr)	45.0 ± 10.9
<55	450 (80.9%)
≥55	106 (19.1%)
Tumor size (cm)	0.7 ± 0.3
≤ 1	460 (82.7%)
≤ 2	96 (17.3%)
Surgery type	
Open	470 (84.5%)
Endoscopic	58 (10.4%)
Robot	28 (5.0%)
Multifocality	
No	486 (87.4%)
Yes	70 (12.6%)
Hashimoto thyroiditis	
No	366 (65.8%)
Yes	190 (34.2%)
Extrathyroidal extension	
No	348 (62.6%)
Yes	208 (37.4%)
Nodal stage	
N0	336 (60.4%)
N1	134 (24.1%)
NX	86 (15.5%)
Recurrence	
No	540 (97.1%)
Yes	16 (2.9%)

### 3.2. Differences in patient characteristics among the N0, N1, and NX stage groups

Patients were divided into 3 groups based on their pathological nodal status, and the relationship between clinicopathological characteristics and pathological nodal status was examined. Table 2 presents the analysis results of the association between clinicopathological characteristics and nodal status. There were no statistically significant differences between the 3 groups in terms of gender, age at diagnosis, tumor size, surgery type, multifocality of cancer, Hashimoto thyroiditis, extrathyroidal extension, nodal stage, and recurrence.

**Table 2:** Clinicopathologic characteristics of patients according to the nodal stage.

Characteristics	N0	N1	NX	*P* value
	(N = 336)	(N = 134)	(N = 86)	
Gender				.076
Female	291 (86.6%)	108 (80.6%)	67 (77.9%)	
Male	45 (13.4%)	26 (19.4%)	19 (22.1%)	
Age (yr)	46.6 ± 10.0	40.7 ± 10.9	45.7 ± 12.4	.094
<55	266 (79.2%)	117 (87.3%)	67 (77.9%)	
≥55	70 (20.8%)	17 (12.7%)	19 (22.1%)	
Tumor size (cm)	0.7 ± 0.3	0.8 ± 0.4	0.6 ± 0.2	.335
≤ 1	286 (85.1%)	104 (77.6%)	70 (81.4%)	
≤ 2	50 (14.9%)	30 (22.4%)	16 (18.6%)	
Surgery type				.081
Open	294 (87.5%)	105 (78.4%)	71 (82.6%)	
Endoscopic	27 (8.0%)	22 (16.4%)	9 (10.5%)	
Robot	15 (4.5%)	7 (5.2%)	6 (7.0%)	
Multifocality of cancer				.223
No	300 (89.3%)	112 (83.6%)	74 (86.0%)	
Yes	36 (10.7%)	22 (16.4%)	12 (14.0%)	
Hashimoto thyroiditis				.051
No	211 (62.8%)	89 (66.4%)	66 (76.7%)	
Yes	125 (37.2%)	45 (33.6%)	20 (23.3%)	
Extrathyroidal extension				.469
No	216 (64.3%)	78 (58.2%)	54 (62.8%)	
Yes	120 (35.7%)	56 (41.8%)	32 (37.2%)	
Recurrence				.151
No	329 (97.9%)	128 (95.5%)	83 (96.5%)	
Yes	7 (2.1%)	6 (4.5%)	3 (3.5%)	

### 3.3. Univariate and multivariate analyses

A univariate analysis was conducted to identify prognostic factors for RFS. Considering all characteristics, we noted no statistically significant differences. Moreover, no significant factors were determined in the subsequent multivariate analysis (Table 3).

**Table 3:** Univariate and multivariate analyses of the recurrence-free survival.

Characteristics		RFS			
		Univariate		Multivariate	
		Hazard ratio (95% CI)	*P* value*	Hazard ratio (95% CI)	*P* value†
Gender	Female	Ref			
	Male	0.34 (0.02–1.70)	.296		
Age (yr)	<55	Ref		Ref	
	≥55	0.95 (0.91–1.00)	.056	0.96 (0.91–1.00)	.077
Tumor size (cm)	≤1	Ref			
	>1	1.11 (0.25–3.52)	.574		
Surgery type	Open	Ref			
	Endoscopic	1.01 (0.35–2.69)	.989		
	Robot	2.25 (0.34–8.51)	.330		
Multifocality	No	Ref		Ref	
	Yes	2.39 (0.65–7.10)	.140	1.94 (0.52–5.94)	.274
Hashimoto thyroiditis	No	Ref		Ref	
	Yes	1.97 (0.71–5.43)	.183	1.83 (0.65–5.11)	.242
Extrathyroidal extension	No	Ref			
	Yes	1.01 (0.34–2.75)	.994		
Nodal stage	N0	Ref			
	N1	2.64 (0.89–7.84)	.275		
	Nx	2.26 (0.58–7.66)	.202		

RFS = recurrence-free survival.

* Kaplan–Meier survival estimates compared by log-rank test.

† Cox proportional hazards model.

### 3.4. RFS analysis

**Figure 2.**
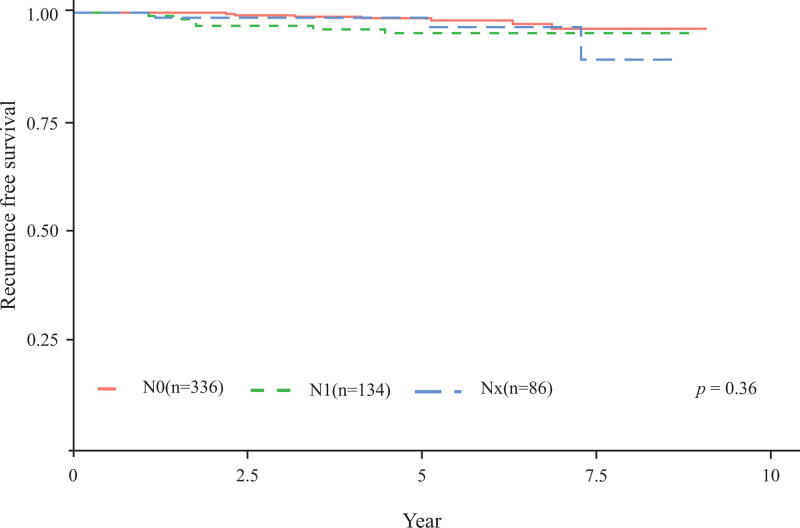
Kaplan–Meier estimates of recurrence-free survival in the N0, N1, and NX groups.

Cancer recurrence occurred in 7, 6, and 3 patients in the N0, N1, and NX groups, respectively. The 5-year RFS rates in the N0, N1, and NX groups were 98.8%, 95.5%, and 97.6%, respectively (*P* = .36, Fig. 2). The contralateral lobe was the most frequent site of cancer recurrence, as identified in 11 patients. Four patients exhibited metastasis of lateral neck LNs and one experienced recurrence at the operation site.

### 3.5. Subgroup analysis of RFS according to the T stage

**Figure 3.**
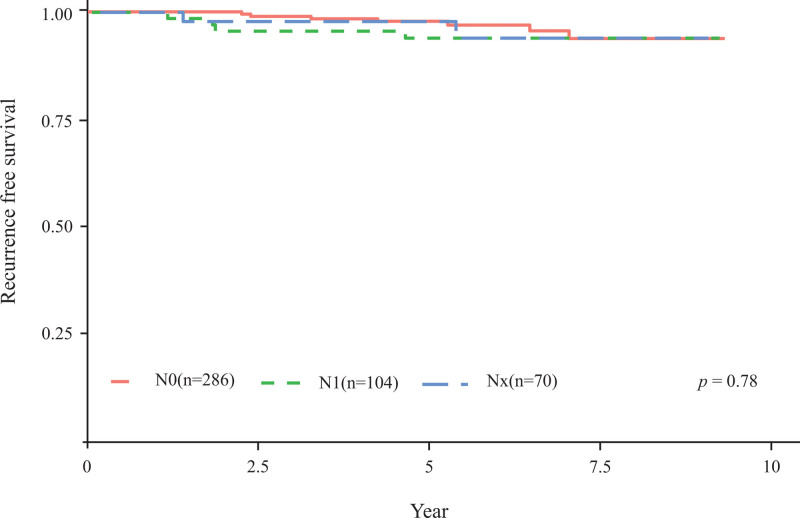
Kaplan–Meier estimates of recurrence-free survival in the N0, N1, and NX groups of patients with papillary thyroid microcarcinoma.

**Figure 4.**
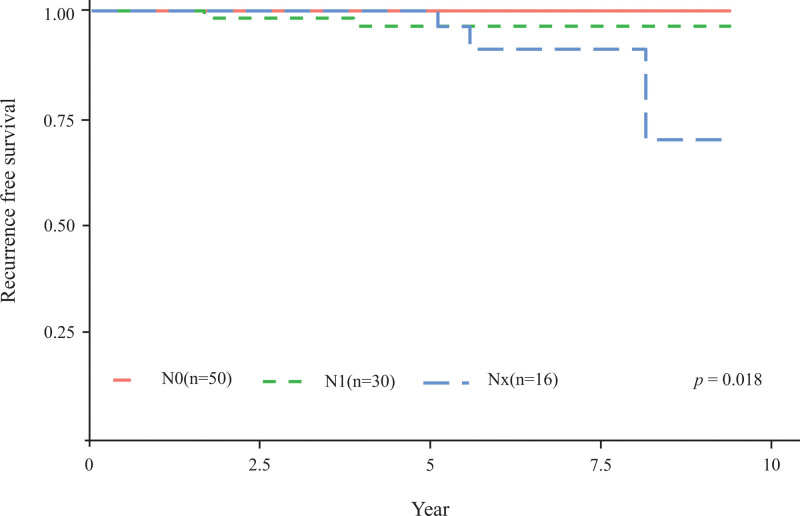
Kaplan–Meier estimates of recurrence-free survival in the N0, N1, and NX groups of patients with T1b stage.

In addition, survival analysis was performed to determine whether nodal status can impact RFS according to tumor size. Considering PTMC, the number of patients in the N0, N1, and NX groups was 286, 104, and 70, with 5-year RFS rates of 98.6%, 96.1%, and 98.5%, respectively (*P* = .78, Fig. 3). Considering the T1b stage (tumor size ˃1 cm and ˂2 cm), the number of patients in the N0, N1, and NX groups was 50, 30 and 16, respectively, and the 5-year RFS rates were 100%, 93.1%, and 93.7%, respectively (*P* = .018, Fig. 4).

## 4. Discussion

Patients with PTC reportedly exhibit a relatively better prognosis than those with other types of cancer, with 20- and 40-year recurrence rates of 6 and 8%, respectively.^[10]^ During a 7-year median follow-up period, patients who underwent thyroid lobectomy for PTC presented a recurrence rate of 3.1%.^[11]^ In the present study, we noted a recurrence rate of 2.9%, and the most common sites of recurrence reported in a prior study^[11]^ and our study were the contralateral lobes and ipsilateral lateral LNs. The risk factors associated with PTC recurrence include extrathyroidal extension, LN metastasis, multifocal tumor, high postoperative thyroid-stimulating hormone, and high postoperative anti-thyroglobulin antibody level.^[10,11]^

Considering the advantages of prophylactic CLND, CLND can decrease central LN recurrence by facilitating the accurate staging of thyroid cancer and reducing the risk of overlooking LN metastases. In a meta-analysis assessing patients who underwent thyroid lobectomy, the authors revealed that those who underwent prophylactic CLND experienced a markedly lower recurrence rate in the central compartment than those who did not undergo prophylactic CLND (0.17% vs 1.78%).^[12]^ Furthermore, increased local recurrence was observed in patients who did not undergo LN dissection, accompanied by bilateral central cervical LN metastases documented in 13 to 50% of cases. In addition, there was no significant difference in the incidence of permanent vocal cord palsy and hypoparathyroidism between the bilateral CLND group and unilateral or no CLND groups, thereby suggesting that bilateral CLND is preferable to complete removal of metastatic LNs.^[13,14]^ Based on previous findings that risk factors for contralateral LN metastasis in patients with unilateral lobe clinically N0 PTC include ipsilateral LN metastasis (*P* < .001) and extrathyroidal extension (*P* = .039), prophylactic CLND may be considered in such cases.^[15]^

In contrast, the incidence of postoperative complications and local recurrence rate did not differ based on prophylactic CLND in patients with clinically N0 PTC subjected to thyroid lobectomy.^[16]^ Additionally, a retrospective study that assessed ˃10,000 patients did not recommend routine prophylactic CLND, as it increases surgical morbidity, such as temporary hypoparathyroidism, instead of lowering locoregional recurrence.^[17]^ Moreover, one study reported that LN metastasis does not increase cancer mortality risk in patients with PTC.^[6]^ And a large-volume retrospective study based on the SEER database, patients without prophylactic CLND had similar thyroid cancer-specific survival compared with those who underwent prophylactic CLND.^[18]^ Based on this perspective, the American Thyroid Association guidelines published in 2016 did not recommend prophylactic CLND for T1- or T2-differentiated thyroid cancers.^[3]^

Based on the findings of a study using 2004 to 2013 SEER data, cancer-specific and all-cause mortality rates were higher in the NX group than those in the N0 group, with no significant difference from those in the N1 group.^[9]^ Furthermore, patients with NX-stage thyroid cancer with distant metastasis have a higher mortality risk (hazard ratio, 1.83) than those with N0-stage tumor regardless of age, race, gender, and tumor size.^[19]^ Hence, differentiated thyroid cancer staged as NX demands an aggressive treatment strategy, as it may unexpectedly lead to a poor prognosis.^[9,19]^ The percentage of NX stage among patients who underwent thyroid lobectomy and prophylactic CLND has rarely been reported. Assessing SEER data, 834 out of 92,447 (0.9%) patients were diagnosed with NX-stage tumor.^[9]^ Another study revealed that 464 out of 3198 patients (14.5%) had an NX-stage tumor.^[19]^ In the present study, 86 out of 556 (15.5%) had an NX-stage tumor. The high percentage of NX stage in our study might be attributable to the surgeon’s preference for avoiding aggressive prophylactic CLND in patients deemed to have low-risk thyroid cancer.

Herein, we examined whether the recurrence rate differs according to pathologic LN status after thyroid lobectomy and prophylactic CLND in patients with clinically N0 PTC. Considering the NX, N0, and N1 groups, there were no significant differences in the recurrence rate and clinicopathological characteristics, including gender, age at diagnosis, tumor size, surgery type, multifocality of cancer, Hashimoto thyroiditis, extrathyroidal extension, and nodal stage. In our study population (T1 thyroid cancer patients), nodal stage, gender, age at diagnosis, tumor size, surgery type, multifocality, Hashimoto thyroiditis, and extrathyroidal extension failed to predict recurrence. However, a subgroup analysis of T1a (≤1 cm) and T1b (>1 cm but ≤2 cm) revealed that recurrence was more common in NX than in N1 and N0 stages in T1b patients.^[8]^ Therefore, although central LN assessment does not predict prognosis in PTMC, more aggressive prophylactic CLND is recommended for T1b, given that a high recurrence is common in the NX stage where central LN assessment is not performed.

Finally, the limitations of the present study need to be addressed. First, this study is a single-institution retrospective study; hence, the findings have limited generalizability. Second, considering that PTC is slow growing, our relatively short follow-up period might be insufficient to adequately assess recurrence. However, despite these limitations, it should be noted that the present study enrolled patients operated on by the same surgeon and assessed the prognosis of NX stage in patients with cancer who underwent lobectomy for low-risk PTC. To accurately determine the prognosis of NX stage in patients who underwent lobectomy for PTC, well-designed multicenter prospective studies with a long follow-up are warranted.

## 5. Conclusion

In conclusion, the prognosis of patients who underwent thyroid lobectomy for PTMC with NX stage did not significantly differ from that of patients with N0 or N1 stage tumors. However, tumor sizes ranging from 1 to 2 cm at the NX stage had a higher recurrence rate than those at N0 or N1 stage, indicating that nodal status should be aggressively rated for tumors ≥ 1 cm in size.

## Author contributions

**Conceptualization:** Ha Rim Ahn, Hyun Jo Youn, Sung Hoo Jung.

**Data curation:** Sang Yull Kang.

**Formal analysis:** Ha Rim Ahn, Sang Yull Kang, Hyun Jo Youn.

**Investigation:** Ha Rim Ahn, Sang Yull Kang.

**Supervision:** Hyun Jo Youn, Sung Hoo Jung.

**Writing – original draft:** Ha Rim Ahn.

**Writing – review & editing:** Hyun Jo Youn.
